# Diffusion Optics Technology (DOT): A Myopia Control Spectacle Lens Based on Contrast Theory

**DOI:** 10.1167/tvst.13.10.42

**Published:** 2024-10-30

**Authors:** Jay Neitz, Maureen Neitz

**Affiliations:** 1 Department of Ophthalmology, University of Washington, Seattle, WA, USA

**Keywords:** myopia, genetics, retinal circuitry, defocus theory, refractive development

## Abstract

Diffusion optics Technology (DOT) myopia control spectacle lenses are based on contrast theory. This innovative theory represents a radical departure from the classical concept of visual deprivation myopia. However, traditional theories have evolved, arriving at remarkably similar solutions for myopia control as the DOT lenses. Nonetheless, contrast theory still represents a departure from mainstream theories. Here, in an effort to resolve discrepancies, we review the science behind contrast theory and compare it to more conventional blur and defocus theories. Finally, we consider the implications of the different theories for the rational design of myopia control solutions.

## Introduction

The contrast theory of myopia led to the development of diffusion optics technology (DOT), a myopia control lens design that has been shown to be effective in clinical trials.^1^ DOT lenses incorporate thousands of light-scattering elements that lower the contrast of images on the retina. Remarkably, the proposal that light-scattering should inhibit myopia rather than stimulate it directly conflicts with the classical concept of deprivation myopia, which has predominated for decades. Blurry images associated with hyperopia during normal childhood development have been thought to stimulate axial elongation; quoting Wallman and Winawer,^2^ “the function of emmetropization is to minimize blur.” Here, we will refer to this standard hyperopic defocus theory as the “blur” theory of myopia to emphasize how it is the opposite of the “contrast” theory of myopia, which holds that the activity of contrast signaling pathways in the retina is the sole driver of eye growth in the retina during refractive development.

Our goal is to reconcile these seemingly conflicting theories, which, as the standard theory has evolved, have converged on two comparable myopia control spectacle lenses, DOT lenses and defocus incorporating multiple segments (DIMS) spectacle lenses.^3^ Whereas DOT lenses incorporate light-scattering elements that reduce the contrast of images across the retina, DIMS lenses incorporate hundreds of tiny microlens segments of 3.5 D addition. In both cases, the power of the base lens is that of the child's distance refractive correction, forming an image that is refracted by the base part of the lens and simultaneously either scattered by the dots (DOT) or differentially refracted by the microlenses (DIMS). Thus, with some light refracted according to the child's correction and some either positively refracted (DIMS) or scattered (DOT), both approaches lower contrast in the peripheral retina.

For the hyperopic eye of a child, whether images are blurred with low contrast or sharply focused and high in contrast depends on the state of accommodation, the distance to the objects being viewed, and whether we are considering the central or peripheral retina. Thus clarifying the relative importance of these factors in refractive development may help resolve seeming conflicts between the hyperopic defocus or “blur” versus the “contrast” theories. More research must be done to fully clarify the role of defocus, contrast, and blur in refractive development. As a step in that direction, we present here a review of contrast theory, highlight features that blur and contrast theory share, point out questions that need to be answered, and consider the implications of the theories for the rational design of myopia control solutions.

**Figure 1.**
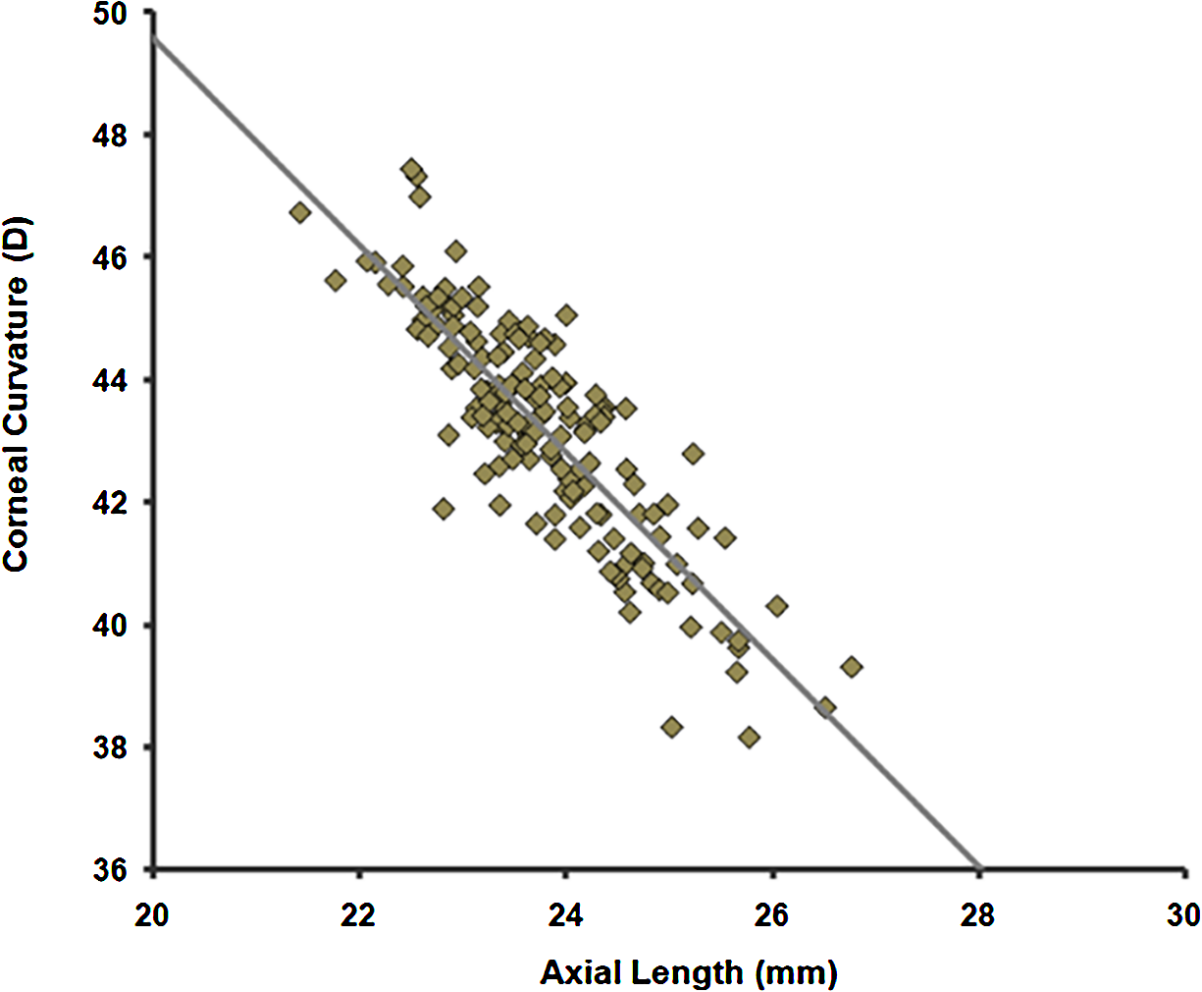
Each data point represents corneal curvature and axial length measurements from an emmetrope measured using a Zeiss IOLMaster (Zeiss, Oberkochen, Germany). There is enormous variability in the eye's optical power, primarily because of differences in corneal curvature. Eyes with more curved corneas have shorter focal lengths. There is a strong inverse correlation (*R*^2^ = 0.7331) between the axial length and corneal curvature as a result of the eye using feedback from the qualities of the images on the retina to control axial growth. These subjects were drawn from a study done in our laboratory in which 373 participants 17 years and over (mean age = 36.3 ± 14.9 years, 45% female, 55% male) were recruited by advertisement through the Optometry and Ophthalmology practices at the Eye Institute of the Medical College of Wisconsin. All research on human subjects followed the tenets of the Declaration of Helsinki and was approved by the IRB at the Medical College of Wisconsin. Respondents with a history of ocular disease, except for refractive error, were excluded from the study. Subjects were divided into four categories: hyperopes (positive SER), emmetropes (no refractive error), low-to-moderate myopes (negative SER < −6 D), and pathological myopes (SER ≥ −6D). Only the emmetrope data is shown.

A fundamental fact of human refractive development that must be explained is illustrated in Figure 1. Adult emmetropic human eyes vary widely in corneal curvature, which is strongly negatively correlated with the eye's axial length. This is because, in normal emmetropization, most young eyes start out too short for their optics, images are formed behind the retina, and eyes grow in length to match their optics. Thus images behind the retina of the unaccommodated eye are associated with eye growth. As the eye grows in axial length, the movement of the retina backward relative to the focal plane of the unaccommodated eye is ultimately associated with inhibiting eye growth. The blur and contrast theories both identify the eye's refractive power as controlling eye growth, which is accelerated in young hyperopic eyes and slowed by myopic defocus. As the eye grows, the change from hyperopia to myopia changes the nature of images on the retina. The questions are as follows: (1) What cues in the retinal image are used to regulate eye growth? (2) In normal refractive development, is blur or contrast driving the growth of the hyperopic eye? (3) Does the emmetropization mechanism have a way of detecting the sign of defocus?

As introduced above, the contrast theory states that the activity of contrast signaling pathways in the retina is the sole driver of eye growth in the retina during refractive development.^4^^,^^5^ This activity can result from clear, well-focused images on the retina, by spurious activity in retinal circuitry that signals contrast caused by genetic mutations or a combination of the two.

## Revolutionary Experimental Discoveries Inspire New Theories

A major commonality between the blur and contrast theories is that they were each formulated in response to revolutionary discoveries related to refractive development. More than 40 years ago, the discoveries by Wiesel and Raviola^6^ that myopia could result from neonatal lid fusion in monkeys, and by Wallman and colleagues^7^ that modest changes in early visual experience could produce extreme myopia resulted in a paradigm shift in thinking about the mechanism responsible for myopia. These discoveries pointed to local retinal processes that use visual signals to regulate growth and refractive state across the retina. Recent groundbreaking discoveries about the genetics of myopia again call for a paradigm shift in thinking about the mechanism responsible for myopia.

Similar to the revolutionary discoveries of the 1970s is the wholly unexpected discovery that a subset of haplotypes of the genes encoding long (L) and middle (M) wavelength-sensitive cone opsins cause exon-skipping during pre-messenger RNA splicing and are associated with extreme myopia.^8^ Like the discovery by Wallman et al.,^7^ what is remarkable about the genetic discoveries is that extreme myopia is associated with seemingly “modest nucleotide changes in the gene.” The original discovery of myopia-causing opsin gene haplotypes was in families with Bornholm eye disease in which the “LVAVA” haplotype of the opsin genes produces by far the largest effect size (>10 D) of any genetic change ever observed in any form of nonsyndromic myopia.^4^

The affected individuals with Bornholm eye disease had both myopia and red-green color vision defects.^9^ Initially, it was assumed that myopia in Bornholm patients was somehow related to color blindness or color vision, but it is not. The splicing defective opsin gene haplotypes that cause myopia are separate and occur equally in people with normal color vision. It is now understood that in people with normal color vision, the myopia-causing haplotypes originally discovered in Bornholm eye disease occur in either the L or M opsin gene but never both. The myopia-causing opsin haplotypes cause a reduced amount of photopigment in the submosaic of cones that express them, as can be seen in adaptive optics scanning laser ophthalmoscopy and optical coherence tomography images,^10^ and they produce smaller responses seen in the electroretinogram,^11^ but otherwise, the affected cones remain viable. This means the retinas of these myopes have cone photoreceptor mosaics made up of normal cones intermixed with cones that are inefficient at absorbing light.

**Figure 2.**
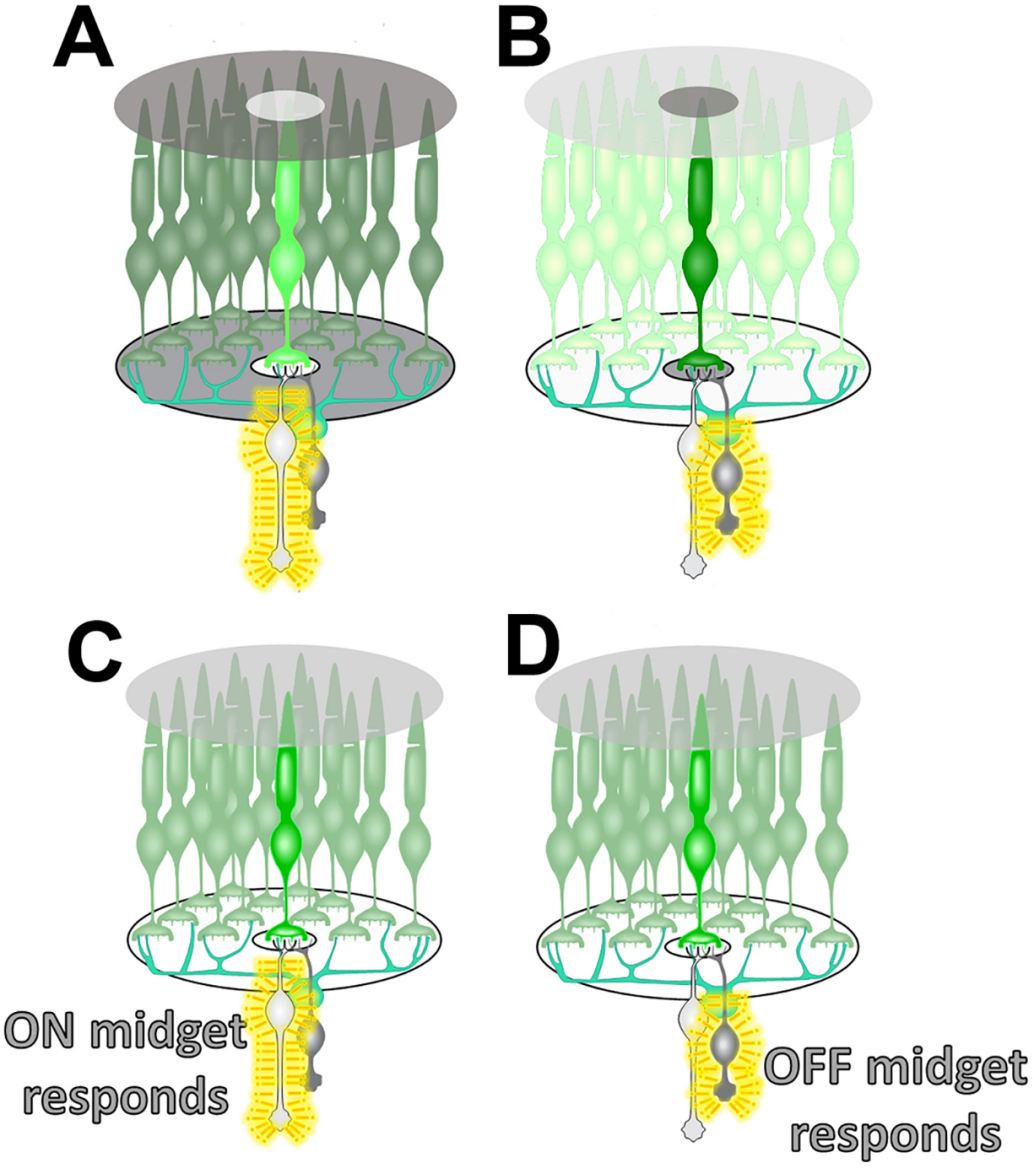
Every L and M cone photoreceptor in the human eye across the entire retina has one ON and one OFF midget bipolar cell. Midget bipolar cells have spatially opponent, center-surround receptive fields, making them contrast detectors. A single cone makes up the center of the receptive field, and its neighbors make up the inhibitory surround mediated by horizontal cells. Normally, when both the center and surround are covered by uniform illumination, the mutually inhibitory center and surround cancel, and there is no response. (**A**) However, when more light from the retinal image falls on the center cone than the surround. The ON bipolar cell is activated–signaling light against a dark background contrast. For example, it could be signaling part of a white letter against a dark background. (**B**) When more light from the retinal image falls on the surrounding cones than the center cone, the OFF bipolar cell is activated, signaling dark against a light background contrast. For example, it could be signaling part of a black letter against a white background. (**C**) If the central cone expresses a normal gene but a submosaic of cones in the surround express the mutation which makes them inefficient at absorbing light, under uniform illumination with no contrast, the cone will absorb more photons than the average of its neighbors and the ON midget bipolar cells will respond, signaling high contrast even in the absence of a high-contrast stimulus. (**D**) Alternatively, If the central cone of a bipolar cell receptive field expresses the mutant gene and is inefficient at absorbing light under uniform illumination with no contrast, it will absorb fewer photons than the average of its neighbors, which are a mixture of normal and mutant cones, and the OFF midget bipolar cells will respond signaling high contrast even in the absence of contrast in the stimulus.

How did the discovery that myopia can be caused by cone mosaics with normal cones intermixed with ones that are inefficient at absorbing light inspire contrast theory? Bipolar cells in the retina carry information from the cones to the inner retina. An essential fact of the human retina is that midget bipolar cells are the primary conduit carrying cone signals in the outer retina and are responsible for high-acuity black-and-white vision. The human visual system compares L and M cones for red-green color vision, but they work together to serve high-acuity black-and-white contrast sensitivity. Midget bipolar cells have center-surround opponent receptive fields (Fig. 2). Every cone in the human retina, even in the periphery, forms the center of one midget ON-bipolar cell and one midget OFF-bipolar cell receptive field.^12^ Balanced center-surround antagonism makes the midget bipolar cells contrast detectors. When the number of photons caught by a cone equals the average number of photons caught by its neighbors, the bipolar cell is not activated, even though its single-cone center absorbs light. When a cone absorbs more photons than the average absorbed by its neighbors, the ON-bipolar cell is activated, signaling light-against-dark contrast in that location in the retinal image (Fig. 2A). Conversely, when a cone absorbs fewer photons than its neighbors in the surrounding area, the OFF-bipolar cell is activated, signaling dark-against-light contrast at that retinal location (Fig. 2B).

Consider an individual with a submosaic of cones that are inefficient at absorbing light because of an exon-skipping mutation. When the receptive field of a bipolar cell is sampling a region of the retinal image with no contrast and is thus uniformly illuminated, if the central cone is normal, the surrounding neighbors will be composed of a mixture of normal and absorption-deficient cones. Therefore the center cone will absorb more photons than the average of its neighbors, and the ON-bipolar cell will be activated (Fig. 2C). At the same time, the receptive field of a bipolar cell with an inefficient cone center will absorb fewer photons than its neighbors, which are a mixture of normal and inefficient cones, and the OFF-bipolar cell will be activated (Fig. 2D). Thus a submosaic of cones that are inefficient at absorbing light will produce spurious activation of both ON- and OFF-bipolar cells—signaling contrast even when there is uniform illumination across the receptive fields.^5^ This spurious contrast signaling in Bornholm eye disease produces extreme myopia (>10 D), which indicates that constitutive spurious contrast signaling at the level of the bipolar cells is a powerful driver of axial elongation. Hence, contrast theory states that the activity of contrast signaling pathways in the retina drives eye growth.

If the genetic results only applied to a rare form of extremely high myopia, there might be a tendency to dismiss the finding as not particularly relevant to common juvenile-onset myopia or to approaches for slowing the progression of myopia. However, there is tremendous haplotype diversity in the cone opsin genes in the normal population.^13^ It has recently been shown that common opsin haplotypes that occur with high frequency in the population are associated with milder splicing defects and are risk factors for common myopia. The large effect size and high frequency in the population make nucleotide polymorphism in the photopigment genes the most significant single gene determinant of common myopia.^4^ About one-quarter of the population has mutations in the photopigment genes that increase the risk of myopia by more than 300%. Thus spurious contrast signaling in the retina caused by common opsin haplotypes is a major contributor to common myopia.

Perhaps the most well-established fact of myopia research is the causal relationship between diffuser wear and myopia. No doubt wearing diffusers, which can reduce contrast on the retina to zero, can cause myopia; thus, under some circumstances, the absence of contrast in images on the retina drives axial growth. At the same time, cones expressing splicing defective opsin gene haplotypes are viable but inefficient at absorbing light. Midget bipolar cell receptive fields that contain a mixture of normal and inefficient cones will signal contrast even when there is no contrast in the retinal image. Individuals with such mutations develop extremely high myopia. Thus, in this case, the activity of contrast signaling pathways in the retina drives axial growth. The fundamental paradox is that both the absence of contrast in images on the retina and the activity of contrast signaling pathways in the retina drive axial growth; however, they do so under entirely different circumstances, which is the key to resolving the paradox. We will return to this paradox and a possible solution later.

## The Problem of the Sign of Defocus

Another commonality between the blur and contrast theories is the recognition that young eyes that need to grow experience hyperopic defocus, while myopic eyes that have grown too long experience myopic defocus. Defocus in either direction results in blur and loss of contrast. It is undeniable that the visual system responds differentially, with hyperopia stimulating growth and myopia slowing it. Proponents of contrast and blur theories have proposed different solutions for how the visual system solves this problem. As a step toward resolving these differences, we clarify the solutions here.

As introduced above, visual deprivation studies indicated that the retina controls axial growth associated with myopia. Visual deprivation involving lid sutures or wearing diffusers reduces contrast on the retina to near zero. Also, hyperopic defocus in young children was expected to reduce contrast. Thus, like visual deprivation, blurry images associated with hyperopia in normal childhood development might stimulate axial elongation, and it was proposed that the “function of emmetropization is to minimize blur.” In short, it has been said that emmetropization is a matter of the eye growing to clarity. Accordingly, many strategies for myopia prevention have focused on corrective lenses that might reduce the amount of blur.

For the proponents of the standard theory, it has been proposed that the emmetropization mechanism must be able to recognize the sign of defocus. One theory is that the eye can recognize stimulus vergence. According to this idea, hyperopic blur stimulates eye growth, whereas myopic blur inhibits eye growth. An alternative is that the emmetropization mechanism uses longitudinal chromatic aberrations (LCA) whereby short wavelengths are in relatively better focus than long wavelengths in the short hyperopic eye, but the relationship switches in the myopic eye for which longer wavelengths are in better focus than short ones. It is proposed that the emmetropization mechanism uses information from LCA to detect the sign of defocus and regulates eye growth accordingly.^14^

Compared to the idea that the eye simply grows to clarity, theories in which the eye recognizes the sign of defocus agree with contrast theory in that myopic defocus (and the resultant reduction in contrast) is associated with slowing/stopping axial growth. The only disagreement is about what happens in the visual system in response to hyperopia. To resolve this disagreement, it is important to know exactly what the two theories propose. *Defocus theory* holds that the emmetropization mechanism recognizes the sign of defocus, and it either drives growth or stops it according to the sign of defocus. *Contrast theory* holds that the hyperopia is compensated by accommodation, bringing images of distant scenery in the peripheral retina into clear focus with high contrast that drives eye growth. Thus both theories agree that the visual system, as a whole, has to be able to respond differently depending on the sign of defocus. However, the contrast theory relies on accommodation to bring distant scenery that fills peripheral vision into clear focus. Then, the emmetropization mechanism, wholly contained within the eye, only needs to react to the time-averaged contrast on the retina, which is proposed to be high for appropriately accommodated hyperopes but low for myopes.

The contrast theory is agnostic about how accommodation works to bring distant scenery into clear focus in the hyperopic eye. All that is important is that contrast theory doesn't require the emmetropization mechanism to recognize the sign of defocus. Instead, taking accommodation into account, very different things happen to images of distant scenery that fill our peripheral retina, depending on whether a person is hyperopic or myopic. For an unaccommodated hyperope, distant scenery is slightly out of focus, and contrast is reduced. However, the hyperope can accommodate to bring distant scenery into clear, sharp high contrast focus on the retina. In contrast, for myopes, distant scenery is already out of focus for the unaccommodated eye, and accommodation makes it more out of focus and lowers contrast. Therefore, in contrast theory, the difference between hyperopic and myopic defocus is that accommodation makes things more clearly focused for the former and less so for the latter. Our eyes are more often accommodated than not, so the time-averaged contrast on the peripheral retina decreases continually as the eye grows from hyperopic to myopic. If contrast in the peripheral retina signals the eye to grow, being hyperopic will make it grow, and when the eye becomes emmetropic, it will stop growing. An important aspect of the theory is that hyperopes can accommodate to bring images in the peripheral retina of distant scenery into clear focus, but they are also unable to fully accommodate to near so that distant scenery is always more in focus than for the emmetrope. Once emmetropia was reached for our paleolithic ancestors, who spent most of their time outdoors, images of distant scenery in the peripheral retina were out-of-focus when the eye was accommodated to nearer objects. For example, when an emmetropic teen is outdoors interacting with a friend, the friend's face fills only a tiny fraction of the retina, while out-of-focus, low-contrast images of distant scenery fill the peripheral retina. Sprague et al.^15^ estimated that, on average, eyes are accommodated to just over a meter, and they are rarely fully far accommodated. Their measurements were made on college student volunteers, but they may also be representative of Paleolithic ancestors, who had to accommodate to navigate obstacles, manipulate objects with their hands, and focus on conspecifics. Thus, for emmetropic eyes, the peripheral retina is most often filled with gentle, low-contrast images of distant, out-of-focus scenery that doesn't drive axial elongation, according to contrast theory.

Whether this scenario is correct still needs to be clarified. Our purpose here is only to explain contrast theory and how it differs from standard theories clearly so the different theories can be evaluated and the differences ultimately resolved by experiments.

## A Note About the Role of Rods

The contrast theory relies on the characteristics of cones. However, the peripheral retina, in which rods vastly outnumber cones, is key in signaling eye elongation. How does contrast theory deal with this fact? According to Curcio et al.,^16^ the human retina contains about 4.6 million cones and 92 million rods, a ratio of 20:1. In the peripheral retina, this ratio becomes dominant outside about 5° of eccentricity, so it is a reasonable question: What is the contribution of the cones when they are so outnumbered by rods? The answer is that contrast theory concerns midget bipolar cells. According to Lee et al.,^17^ in the periphery, there are about 18 rods for every rod bipolar cell. This convergence is part of what gives the rod system its high sensitivity compared to the cones. In contrast, the cones diverge so that there are two cone midget bipolar cells, one ON and one OFF, for every cone. So, at the level of the bipolar cells in the periphery, there are about twice as many midget cone bipolar cells as rod bipolar cells. Moreover, the rods are saturated at normal daytime light levels, so they do not contribute to vision. These factors combined make our daytime vision at the level of bipolar cells based primarily on cones, even in the periphery. This is not to say that rods don't play a role. It is just that the current evidence for contrast theory doesn't include any information about rod involvement.

## Why Do Some Children Become Myopic Whereas Others Don't?

Proponents of both the contrast and defocus/blur theory agree that why some children become myopic and others don't must be explained. Theories must be evaluated based on their ability to explain and predict. The contrast theory holds that increased activity in the contrast pathways signals the eye to grow, and reduced contrast signaling reduces eye growth such that separate “start” and “stop” signals are not required to explain the etiology of myopia.^18^ Finally, contrast theory holds that contrast signaling is the final common pathway responsible for both genetic and environmental contributions to myopia.

Accordingly, the spurious signaling in the contrast pathway caused by various genetic mutations plus the abnormal exposure to high contrast images on the peripheral retina produced by printed materials and video displays of all types, including television, computers, laptops, and tablets, are combined to determine a person's refractive error. Thus genetic mutations that cause spurious contrast signaling in the retinal contrast pathways are an explanatory factor of why some children become myopic, with about 25% having mutations in the photopigment genes that increase the risk of myopia by more than 300%.^4^^,^^5^ Several other familial factors have been implicated in myopia. For example, it is said that children inherit myopic behaviors from their myopic parents: more books in the home, more reading, and less time outside. This is consistent with the combined effects of spurious signaling and exposure to myopiagenic stimuli causing myopia.

For two reasons, the discovery of splicing defective opsin genes as an important risk factor in myopia development is a major advance in understanding myopia's etiology. First, even though meta-analysis of more than one-half million individuals subject to genome-wide association studies (GWAS) have identified nearly 900 single nucleotide polymorphisms, they only account for 18.4% of the heritability of myopia,^19^ leaving 81.6% unexplained, and now identifying splicing defective opsin genes as myopia's single major genetic determinant explains a significant fraction of the previously unexplained heritability. Why didn't GWAS identify the opsin polymorphisms as being associated with myopia, much less the major genetic determinant? This is because the splicing defects arise from a recombination mechanism that exchanges nucleotides between the long (L) and middle (M) wavelength-sensitive opsin genes.^8^ These mutations occur at an extremely high rate, repeatedly generating the same exon 3 haplotypes in all different genetic backgrounds. They are thus invisible to GWAS, which relies on identifying disease-causing genes by virtue of their having arisen on a particular background. The second reason the identification of these defective opsin genes is a major advance is that this is the only case in which the gene causing common myopia has been linked to the exact physiological change responsible—that is, reduced expression of photopigment in a submosaic of cones leading to spurious activity of midget bipolar cells, which make up more than 80% of bipolar cells in the human retina. This reveals both the cause of myopia and illuminates a path to effectively preventing it.

In contrast, the question of why some children become myopic and how to prevent it has been a challenge for blur theories of myopia. It has long been recognized that having myopic parents is a risk factor for myopia. The blur theory proponents have proposed why blur might persist more in children who develop myopia. For example, it has been suggested that myopic children might have residual peripheral hyperopic defocus even when the fovea has reached emmetropia, so eye growth driven by the peripheral retina results in myopia. However, the idea that relative peripheral hyperopia predicts the development and progression of myopia in children has been challenged in some studies.^20^

## What Are the Retinal Circuits Responsible for Signaling Eye Growth?

Contrast theory specifies the exact retinal circuitry in the human eye responsible for the contrast detection that mediates eye growth—the midget bipolar cells—and it proposes the precise mechanism of how the progression of the eye from hyperopia to myopia during development gradually modulates the activity of the midget bipolar cells which signal contrast to regulate eye growth. Moreover, the exact mechanism of how myopia-causing genes disrupt the normal function of the midget bipolar cells has been proposed as part of contrast theory.

Circuitry for detecting the sign of defocus as part of the emmetropization mechanism is not required for contrast theory since it relies on the idea that accommodation can bring distant scenery into clear, high-contrast focus for the hyperopic eye but accommodation lowers the contrast of images of distant objects in the peripheral retina of myopes.

On the other hand, results from Swiatczak and Schaeffel^14^ using a color display have been reported to be consistent with LCA being used as a cue for detecting the sign of defocus as part of the emmetropization mechanism. However, evidence for the appropriate retinal circuitry is lacking. In Swiatczak and Schaeffel's^14^ “red in focus” condition, the red channel was unfiltered, while green and blue were blurred according to the human LCA function. In humans, midget cells are the only bipolar cells capable of differentially responding to red light. However, they typically have L versus M center-surround receptive fields that respond to both uniform (i.e., blurred) areas of red light and highly focused patterns of red light, so they cannot tell blurred from a focused red light. This makes it uncertain whether any circuitry capable of distinguishing blurred from in-focus red light exists in the retina.

Midgets that make up more than 80% of the bipolar cells in the retina and their circuitry are incapable of playing a role in detecting the sign of defocus. However, with regard to contrast theory, even though midget bipolar cells are L versus M cone opponent, the M-center and L-center cells work together to serve black-and-white vision,^21^ and they are solely responsible for mediating high-acuity achromatic contrast. Disruption of the appropriate contrast signaling in midget bipolar cells in individuals with cone opsin polymorphisms, as proposed in contrast theory, produces refractive errors of >10 D. This is powerful evidence that achromatic contrast signals carried by the midget bipolar cells drive eye growth during emmetropization, as proposed by contrast theory.

With regard to sign of defocus theories, S-cones have been proposed to play a role.^22^ Indeed Gawne et al.^23^ argue that the spacing of the short-wavelength sensitive cones in humans is sufficient for them, in conjunction with the L and M cones to use chromatic signals to guide emmetropization. However, the S-cone bipolar cells are S versus (L + M) cone opponent in the human eye,^24^^–^^26^ and they respond to both uniform (or blurred) areas of blue light and to focused patterns of blue, so they cannot serve to differentiate blue in focus versus blurred. Finally, if S-cones were important for emmetropization, we would expect people with tritan color vision defects to have a high incidence of refractive errors, but there is no evidence for this.^27^ The most important point is that theories about how the emmetropization mechanism might use LCA are proposed specifically based on the premise from defocus theory that it has to detect the sign of defocus. Contrast theory does not require that the emmetropization mechanism be able to detect the sign of defocus because this is handled by accommodation. This is consistent with the absence of retinal circuitry in the human retina capable of encoding cone-type-specific contrast.

The midget system is central to contrast theory, but it is not present in laboratory animals commonly used in myopia studies. Thus it is uncertain what mechanisms responsible for refractive development humans share with lower mammals and birds. Vertebrates share a great deal of common evolution, so some of the fundamental aspects of refractive development must be shared, and many lessons learned from animal studies must apply to humans. On the other hand, human eyes are greatly different from birds and lower mammals, so some aspects of emmetropization are likely to be different. The relevance of animal studies to human myopia needs to be clarified. In the meantime, contrast theory is based on results from primates and humans, in particular, ensuring they are relevant to therapies for human myopia.

## Reconciling Contrast Theory With Form Deprivation Myopia

There is no doubt that under conventional laboratory conditions, strong diffusers that drastically lower contrast are associated with axial growth of the eye. This may be the most well-established fact of myopia research, and it is the single most compelling result in favor of blur theory. However, recent findings require us to modify our hypotheses about contrast and myopia. Smith and colleagues^28^ showed that when nonhuman primates wore strong diffusers under conditions where auxiliary lighting raised the light levels to ∼25,000 lux during the middle of the day, most animals tested developed form-deprivation hyperopia.

The finding that diffusers worn under light levels more equivalent to full daylight outdoors are associated with hyperopia in primates signals a paradigm shift away from the classical concept of deprivation myopia. Strikingly, the high light-level results are consistent with contrast theory. Because our evolutionary ancestors were outdoor dwellers, the high luminance results may be more relevant to understanding emmetropization mechanisms than those obtained under unnatural low-light laboratory conditions.

The reason why diffusers are associated with myopia when worn at low light levels but hyperopia when worn at high light levels remains to be clarified. Contrast adaptation in response to altered image quality may be an important factor. There are contrast adaptation mechanisms in the retina that increase the gain of contrast detectors when the contrast on the retina is reduced.^29^ It is also true that cone photoreceptors and bipolar cell pathways are noisy.^30^ Under the very low–contrast conditions produced by strong diffusers, the contrast gain may be increased such that intrinsic noise in the retina drives spurious contrast signals. Thus, because of greatly amplified intrinsic noise, very low contrast may paradoxically drive contrast signaling. Noise in the visual pathway is reduced at high light levels, possibly explaining why diffusers worn at high light levels are associated with hyperopia, as predicted by the contrast theory.

On the other hand, contrast adaptation would also be expected to reduce the spurious contrast signals caused by opsin mutations. Thus, it is important that the relative effects of contrast gain be clarified in considering both form deprivation myopia and contrast theory. Along a similar line, form-deprivation myopia in monkeys is a graded phenomenon and can be triggered by a modest degree of chronic image degradation.^31^ However, this is under low-light laboratory conditions, and presumably it would not hold at higher light levels. Under higher light levels similar to the outdoors, weak diffusers would be expected to slow axial growth as demonstrated by strong diffusers at high light levels.^31^ Moreover, clearly, the very weakest diffuser associated with myopia at low light levels is very different from the DOT spectacle lenses that provide good visual acuity and distinct images while slowing myopia progression. Still, the exact parameters for diffusers that cause myopia versus therapeutic spectacle lenses that lower contrast and slow myopia progression need to be clarified.

Visual deprivation myopia results are a cornerstone of blur theory. However, myopia associated with visual deprivation at low levels in the laboratory may be the exception rather than the rule. If the high luminance form-deprivation hyperopia observed by Smith and colleagues^31^ is more relevant to human physiology than the idea myopia treatment should minimize blur, then the explanatory and predictive power of contrast theory may be the key to developing effective treatments for myopia.

The theory that activity in the contrast signaling pathways in the retina is the sole driver of axial growth during emmetropization is based on several premises. That (1) peripheral visual signals dominate refractive development, (2) in the natural environment in which emmetropization mechanisms evolved, images of distant scenery often fill the peripheral retina, and (3) young eyes are “far-sighted,” meaning that they can typically bring images of distant scenery into clear focus via accommodation.

Fundamental claims of contrast theory remain to be clarified. These include the following: (1) The claim that accommodation compensates for hyperopic blur in young children, bringing distant scenery into sharp, high-contrast focus. Accordingly, the difference between hyperopic and myopic defocus is that accommodation reduces hyperopic defocus and increases contrast on the retina, whereas accommodation increases myopic defocus, lowering contrast. (2) The claim that eyes are accommodated to some degree most of the time, and as the eye grows from hyperopia through emmetropia to myopia, accommodation will progressively reduce the amount of time clear images are formed on the peripheral retina. Thus the time-averaged reduction of peripheral contrast in the natural world will result in slowing eye growth as emmetropia is reached. Here, we have explained contrast theory, including principal aspects that need to be clarified so they can be tested experimentally in the future. Ultimately, lessons learned can be applied to better understand the mechanisms underlying current myopia solutions and to develop the next generation of treatment options.

## Implications of Contrast Theory for the Development of Optical Solutions to Control Myopia

Animal studies have long shown that imposing myopic defocus in the periphery (thus lowering contrast on the retina) can slow axial growth.^32^ Moreover, increasing the positive power in myopia control lenses over what might compensate for any peripheral hyperopia (e.g., 3 D)^33^ has been shown to be more effective in controlling axial growth. Thus there can be little doubt that lowering contrast by myopic defocus reduces eye growth, as predicted by the contrast theory.

Earlier, we introduced that DIMS^3^ spectacle lenses employ hundreds of tiny microlens segments of 3.5 D addition on a “background” of single vision correction throughout the periphery of the lens. This amount of addition is much greater than would be expected to compensate for any peripheral hyperopia, instead focusing light in front of the retina while at the same time focusing light transmitted through the single correction portions at a different plane. Other spectacle lens myopia control designs, including H.A.L.T.^34^ and CARE^35^ lenses, have followed a similar approach, with the CARE lenses adding a full 8.00 D of cylinder power to the base power of the occupied areas. Thus modern solutions to myopia progression depart from the old idea of producing clear, well-focused, high-contrast images on the retina. These solutions share, in common with DOT lenses, that they lower the contrast of images on the retina, consistent with the contrast theory of myopia. However, other lens solutions also alter the vergence of light at the retina, and it can be argued that the myopic defocus provided by the positive addition, not just the lowering of contrast, is important. A body of research suggests that there may be more than one signal driver, and it may be possible that contrast and defocus provide some nonidentical cues.

Contrast theory holds that the change in contrast that occurs during emmetropization is used by the eye to gain information about how well images are focused. High-acuity vision in humans is based on midget bipolar cells that are contrast detectors, consistent with contrast theory. However, no known neurons in the eye can distinguish defocus from a change in contrast produced by scattering as required by defocus theories. Nonetheless, the importance of myopic defocus versus just lowering contrast remains to be resolved.

If contrast theory is correct, it offers clear predictions for the rational design of myopia control lenses that can maximize both efficacy and tolerability. These advantages are evident in a comparison between Diffusion Optics Technology (DOT) lenses and the designs employing positive addition segments to the peripheral areas of the lens. First, DOT lenses produce good visual acuity and distinct images across the lens, created by the single correction background of the lens. A uniform plane of scattered light is superimposed on images formed by the background lens, lowering contrast slightly but uniformly. Thus good vision is provided by the lens no matter what region the child is looking through. The images are sharp; they are just slightly lower in contrast. This differs from the positive power of microlenses that distort and blur the image, making it more difficult to see clearly through the “treatment zone.” Second, the DOT lenses' treatment zone covers the entire lens except for the small, clear central area with no dots. The entire lens is designed to be looked through. The tiny clear center is only provided for the rare occasions when the child wants to view a small distant object at full contrast. In typical visual behaviors such as reading, children can make normal eye movements and see clearly, looking through all parts of the lens as they move their eyes.

Finally, in the mission to reduce the incidence and severity of myopia as much as possible, contrast theory makes precise predictions about the potential of effectively treating pre-myopic children. Contrast theory holds that axial growth is driven by activity in contrast pathways produced by a combination of spurious signaling in the contrast pathways caused by various genetic mutations and exposure to myopiagenic stimuli. Because children with mutations that produce spurious signals in the contrast pathway are at a higher risk for developing myopia, it makes sense to have pre-myopic children start wearing DOT lenses without any refractive correction. The lenses do not control the spurious signaling in the contrast pathways caused by their genetics; instead, they reduce the total burden of contrast signaling in the retina. By starting early, along with managing myopia risk factors, it may be possible to proactively manage myopia in some children. For others, wearing the lenses starting before myopia develops may reduce refractive errors to a minimum, preventing the long-term vision problems associated with larger refractive errors.

Many aspects of the mechanisms responsible for myopia have yet to be clarified. Contrast theory has emerged as an alternative to theories in which the emmetropization mechanism can recognize the sign of defocus based on revolutionary discoveries that mutations in genes encoding the cone opsins cause spurious contrast signaling and are associated with both non-syndromic high myopia and common myopia. Theories in which the eye recognizes the sign of defocus require the eye to detect vergence signals or information from longitudinal chromatic aberration, while contrast theory proposes that the activity of contrast signaling pathways in the retina is the sole driver of eye growth during refractive development. The alternative theories make very different predictions about the most effective measures for controlling myopia; therefore future experiments to test those predictions will be able to distinguish between them or suggest a compromise.
